# Understanding Willingness to Share SBEDOH Information: Cultural Sensitivity and Community Engagement in ADRD Research

**DOI:** 10.1002/alz.092391

**Published:** 2025-01-09

**Authors:** Larry D. Adams, Farid Rajabli, Mary E Douglas, Jose Javier Sanchez, Rhoda Moise, Michael L. Cuccaro, Margaret A Pericak‐Vance, Azizi A Seixas

**Affiliations:** ^1^ John P. Hussman Institute for Human Genomics, University of Miami Miller School of Medicine, Miami, FL USA; ^2^ Dr. John T. Macdonald Foundation Department of Human Genetics, University of Miami Miller School of Medicine, Miami, FL USA; ^3^ John P. Hussman Institute for Human Genomics, Miami, FL USA; ^4^ Department of Psychiatry and Behavioral Sciences, University of Miami Miller School of Medicine, Miami, FL USA; ^5^ 1501 NW 10th Avenue, Miami, FL USA; ^6^ Department of Informatics and Health Science Data, University of Miami Miller School of Medicine, Miami, FL USA

## Abstract

**Background:**

Understanding social, behavioral, and environmental determinants of health (SBEDOH) are key in identifying modifiable risk factors for Alzheimer’s Disease and related Dementias (ADRD), particularly the disproportionate burden seen in minorities. However, a lack of standardized SBEDOH methods exists with current methods limited in scope and cultural sensitivity (i.e. can be used across different settings and populations and devoid of stigma). To address these gaps, we created a SBEDOH survey and conducted focus groups in Black American (BA) and Hispanic/Latino (H/L) participants to determine the culturally sensitivity and how best to communicate the survey benefits to diverse groups.

**Method:**

Focus groups were conducted, engaging 11 BA and 15 (H/L) individuals, using semi‐structured interviews to elicit in‐depth discussions around the cultural sensitivity of SBEDOH items (56 questions, across 13 SBEDOH domains). Sessions were transcribed verbatim, and thematic analysis performed using *NVivo* software. Two primary coders analyzed data, with a third coder resolving discrepancies. The semi‐structured nature of the interviews facilitated flexible dialogue, probing deeper to responses, enriching the data collected for cultural nuances.

**Result:**

We identified nine cultural sensitivity themes: initial reactions, participation interest, impact perception of information sharing, survey benefits communication, research engagement, tangible benefits, potential misuse of shared data, emotional reactions, and attitudes towards SBEDOH. BA’s reluctance was linked to historical mistrust (e.g. slavery), while H/L’s were about privacy. Both groups noted the importance of clarity on the survey’s benefits to their communities and research at‐large. Readiness to participate was contingent upon careful consideration of these aspects to ensure cultural sensitivity and trust‐building. Both groups emphasized the expectation that information be relayed by culturally responsible professional and SBEDOH should come with guidance on available social care services, highlighting the importance of a reciprocal relationship for sharing sensitive information.

**Conclusion:**

This study affirms that when approached with cultural sensitivity and clear communication, BA and H/L individuals are receptive to sharing SBEDOH information. The significance of culturally attuned methods and articulation of direct community benefits are critical to success, underscoring the need for an environment that fosters connectivity and trust resulting in enhanced participation in ADRD research.

